# Effects of whole life exposure to Bisphenol A or 17α-ethinyl estradiol in uterus of nulligravida CD1 mice

**DOI:** 10.1016/j.dib.2015.10.034

**Published:** 2015-11-03

**Authors:** Jessica A. Kendziorski, Scott M. Belcher

**Affiliations:** Department of Pharmacology and Cell Biophysics, University of Cincinnati, Cincinnati, OH 45267, USA

**Keywords:** BPA, Estrogen, EDC, endocrine disruption, Collagen, Fibrosis, Immune, Macrophage

## Abstract

Bisphenol A (BPA) is an endocrine disrupting chemical (EDC) with known estrogenic activity. Exposure to BPA in adult mice was shown previously to increase uterine pathology with associated alterations in the immune response and fibrosis. Reported here are uterine histopathology findings from CD1 mice exposed to BPA or 17α-ethinyl estradiol at multiple doses from conception through postnatal day 90. Along with uterine pathology, impacts of exposure on collagen accumulation and F4/80 positive macrophage numbers, as an indicator of immune response in the endometrium and myometrium, are presented. These companion data are from offspring (F1) of the dams analyzed for effects of adult exposures published in the *Reproductive Toxicology* manuscript titled “*Strain-Specific Induction of Endometrial Periglandular Fibrosis in Mice Exposed during Adulthood to the Endocrine Disrupting Chemical Bisphenol A*” (doi: 10.1016/j.reprotox.2015.08.001).

**Specifications table**

**Table:** 

Subject area	Reproductive biology
More specific subject area	Reproductive toxicology, endocrine disruptors
Type of data	Table, graph and figures
How data was acquired	Histological and immunohistochemical stains: hematoxylin & eosin (H&E), picrosirius red, and anti-F4/80 (ab6640, Abcam)
Bright field and circular polarization microscopy of stained tissue sections using Nikon 55i or 80i microscope with polarized light attachment and Digital Sight Software
Data format	Primary data, quantified and analyzed graphs
Experimental factors	Whole life BPA or EE exposure: preconception to PND90; oral route of administration; paraffin embedded and sectioned uterus
Experimental features	Assessment of uterine pathology and alterations in fibrosis/collagen accumulation and immune response in comparison to unexposed control; dose response for 5 BPA and 3 EE dose groups
Data source location	Cincinnati, OH, USA
Data accessibility	Data are present with this article

**Value of the data**

• The presented analysis for offspring (F1) is complementary to previously published data for F0 females (1).
• The findings are useful for comparative analysis of exposure timing for determining the impact(s) of estrogen-like EDCs in uterus across the life span.
• The presented analyses of three different time points inform on how these uterine endpoints change with age.
• The dose response analysis affords comparative assessment of uterus estrogen-sensitivity and identification of differences between strains and life-stages

## 1 Data

**Fig. 1:**
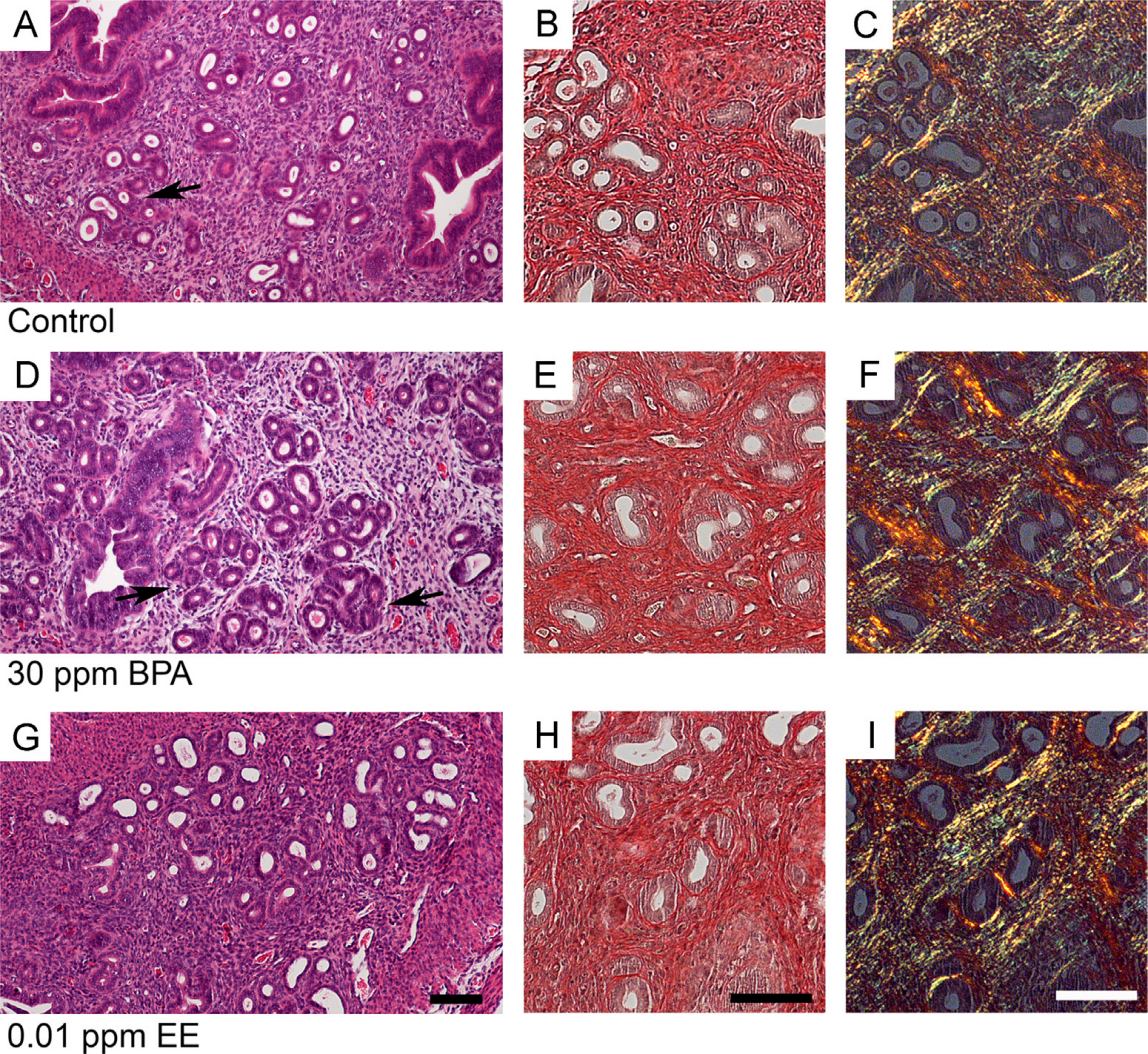
Examples of uterine morphology and collagen accumulation in uterus of control (A–C), 30 ppm BPA (D–F) or 0.01 ppm EE (G–I) exposure groups at PND90. Shown are photomicrographs of H&E and picrosirius red-stained uterine sections from similar uterine locations in each panel. In panel D a region of uterus with abnormal pathology is shown. Arrows are indicating an example of a developing gland nest structure. These structures were observed at a low frequency in all groups. Exposure related changes in picrosirius red-staining indicating alterations of collagen accumulation were not apparent. Scale bars represent 50 µm.

**Fig. 2:**
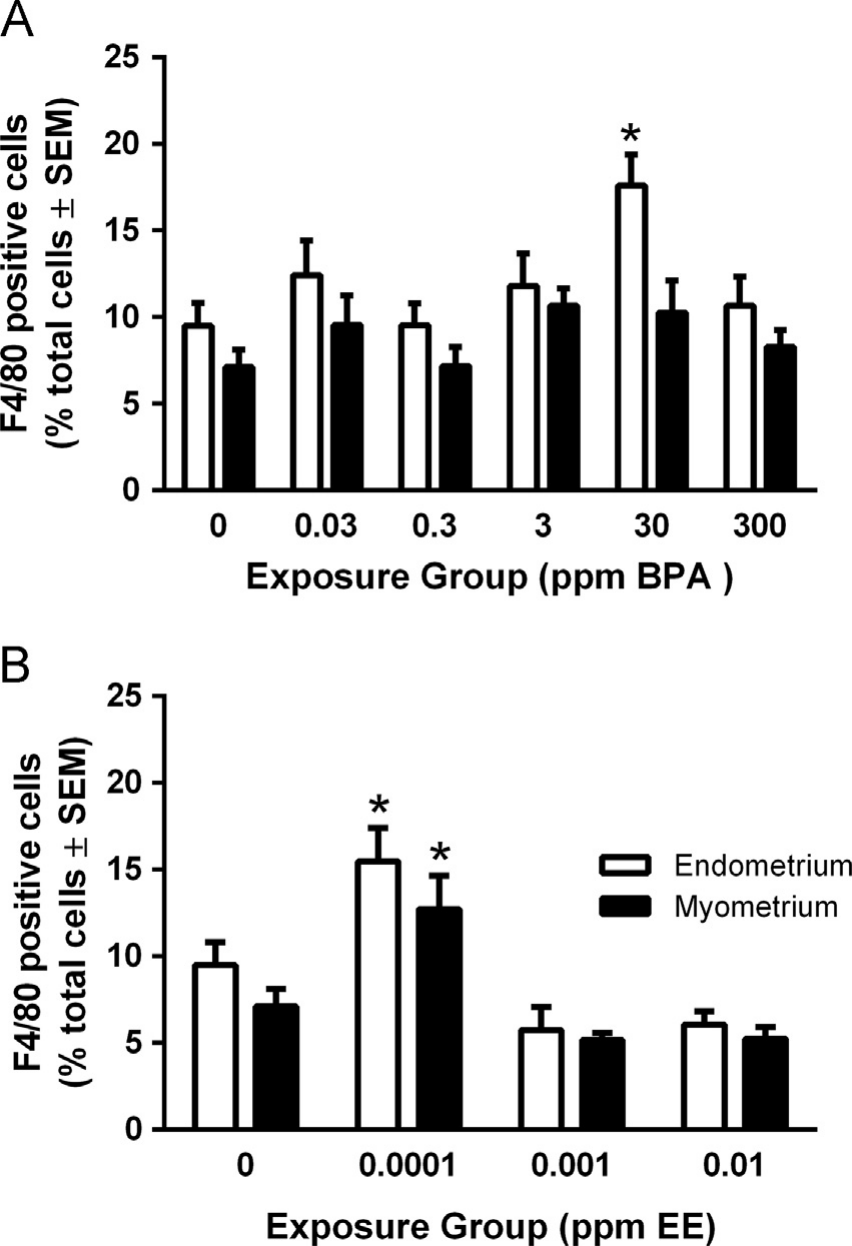
Quantification of F4/80-positive cells in BPA- or EE-exposed CD1 offspring. In CD1 offspring, endometrial (white bars) or myometrial (black bars) F4/80-positive cells were counted in BPA- (A) or EE-exposed (B) mice. Endometrial and myometrial F4/80-positive cells were quantified separately and normalized to either stromal or smooth muscle cells respectively. F4/80-positive and total cells were counted by an observer blinded to exposure group. Values indicated are the percent of F4/80-positive cells per animal and error bars that represent the SEM. The level of statistical significance between values was assessed for each compound using a two-way ANOVA and Bonferroni post-hoc test comparing effects of dose.

The results in Table 1 are pathology analysis presenting the incidence of distended glands, uterine cysts, and the density of gland nests in the endometrium for control and each BPA and EE exposure group at PND 21, 49, and 90. Significant age-related increases in distended glands were observed in most exposure groups. As expected for the CD1 strain, the density of gland nests was very low in all groups at PND90. No significant exposure related differences in gland nest densities were detected for BPA [F(5, 62)=1.843, *p*=.1176] or EE [F(3, 30)=0.8388, *p*=.483]. Examples of photomicrographs for H&E and picrosirius red-stained uterine sections from control, 30 ppm BPA, and 0.01 ppm EE exposure groups are presented in Fig. 1. The percentage of F4/80-positive cells was used as a marker of immune response and was quantified for CD1 mice at PND90 in control and each BPA or EE exposure group (Fig. 2). Two-way ANOVA of F4/80 positive macrophage numbers indicated significant main effects of BPA dose [F(5, 70)=2.876, *p*=.0203)] and uterine structure [F(1, 70)=10.13, *p*=.0022] with no significant interaction between factors [F(5, 70)=0.7783, *p*=.5687] and for EE exposure, there was a significant main effect for dose [F(3, 34)=18.57, *p*<.0001]. Bonferroni׳s multiple comparisons test indicated that compared to control, the percentage of F4/80 immunopositive cells in the 30 ppm BPA exposure group significantly increased (*p*=.0185). A significant increase in F4/80 cells was also detected in both endometrium (*p*=.0130) and myometrium (*p*=.0220) for the lowest dose of EE.

## 2 Experimental design, materials and methods

### 2.1 Animal husbandry and necropsy

All animal procedures were performed in accordance with protocols approved by the University of Cincinnati Institutional Animal Care and Use Committee and followed recommendations of the Panel on Euthanasia of the American Veterinary Medical Association. Mice were acclimated to a polycarbonate-free caging system that limited contamination from exogenous estrogenic compounds. The composition of the control and test diets used were described in detail previously [1], [2], [3]. Dosing was experimentally designed to span a range of exposures below the acceptable oral limit of BPA for humans (50 µg/kg body weight/day) and approach the no observed adverse effect level (NOAEL) for BPA of 50000 µg/kg/day [4], [5], [6]. Diets containing BPA at known concentrations (0.03, 0.3, 3, 30, and 300 ppm) resulted in doses of ~4, 40, 400, 4000, and 40000 µg/kg/day, and concentrations of EE (0.0001, 0.001, and 0.01 ppm) resulted in doses of ~0.02, 0.2, and 1 µg/kg/day. Control and experimental diet was fed to sires, dams and analyzed offspring *ad libitum* throughout their lifespan. Dams and sires were exposed to BPA or EE for two weeks prior to mating. To best replicate the human exposure throughout the differing life stages, offspring exposure occurred through maternal consumption of test diet during the fetal and lactation periods, with uninterrupted dietary exposure continuing through sacrifice at PND90. The female offspring (F1) were separated from dams at PND 21 and housed by exposure group 5 per cage. The nulligravida females analyzed here were sacrificed in estrus based on a cytological analysis of vaginal lavage, with staging confirmed based on uterus morphology. Full details for the study can be found in Kendig et al. [2].

### 2.2 Histology and immunohistochemistry

Tissues were embedded, sectioned, and stained with H&E as previously described [1], [2], [7]. Stained sections were examined on a Nikon Eclipse 55i microscope using a DS-Fi1 CCD camera controlled with Digital Sight Software (Nikon; Melville, NY). Uterine tissue from CD1 offspring at PND21, 49, and 90 was examined for distended glands, cysts, and gland nests as described [1].

Immunohistochemistry using a rat monoclonal anti-F4/80 antibody (C1:A3-1; 4 µg/mL (1:250); ab6640, Abcam; Cambridge, MA) was performed to quantitatively determine alterations in macrophage number as an indicator of immune response in the uterus of CD1 offspring at PND90 as previously described [1], [7]. Five random 40x fields of the myometrium and five random 40x fields of the endometrium were collected from a single longitudinal section of uterus for each animal and the total cell number and number of immunopositive cells per field was counted. To account for possible region specific differences in cellularity and differences in luminal area, the mean percentage of immunopositive cells in the myometrium and the endometrium for each section was calculated and taken to be representative of the number of F4/80 positive cells for that individual. Paraffin-embedded uterine sections were stained with Picrosirius Red (Polysciences; Warrington, PA) to visualize total collagen (red) with bright field illumination and by polarization birefringence to assess the thickness and packing density of collagen fibers [8], [9], [10]. Staining and analysis procedures were previously described in detail [1]. Stained sections were examined on a Nikon Eclipse 80i microscope equipped with a polarized light attachment and a DS-Fi1 CCD camera controlled with Digital Sight Software (Nikon; Melville, NY). Final figures were generated using Adobe Photoshop.

### 2.3 Statistical analysis

The observer was blinded to exposure group. The litter was used as the statistical unit; one animal was randomly selected from each litter as a representative of that litter. Pathology was assessed with statistical differences analyzed using Fisher׳s exact test or *χ*^2^ test for trend when assessing across age. Gland nest density was analyzed using a one-way ANOVA and Dunnett’s multiple comparison test. The percentage of F4/80-positive cells was analyzed using a two-way ANOVA and Bonferroni post-hoc test, in which exposure and location in uterus (endometrium or myometrium) were used as the main effects. Outliers were removed based on Grubb’s test. Significance between differences in values was defined as *p*<0.05. All data was analyzed using GraphPad Prism^®^ v5/v6 software (GraphPad; La Jolla, California).

## Figures and Tables

**Table 1:** Uterine Pathology in CD1 Mice.

	**Exposure group:**			**BPA (ppm)**			**EE (ppm)**		
	**0**	**0.03**	**0.3**	**3**	**30**	**300**	**0.0001**	**0.001**	**0.01**
**PND 21**									
*n*=	5	6	5	7	7	5	5	6	5
Distended glands	0 (0%)	1 (17%)	0 (0%)	0 (0%)	1 (14%)	1 (20%)	1 (20%)	0 (0%)	2 (40%)
Cysts	0 (0%)	0 (0%)	0 (0%)	0 (0%)	0 (0%)	0 (0%)	0 (0%)	0 (0%)	0 (0%)
**PND 49**									
*n*=	9	8	11	8	5	10	10	5	7
Distended glands	2 (22%)	1 (13%)	4 (36%)	5 (63%)	2 (40%)	2 (20%)	2 (20%)	1 (20%)	2 (29%)
Cysts	0 (0%)	0 (0%)	1 (9%)	0 (0%)	0 (0%)	0 (0%)	0 (0%)	1 (20%)	0 (0%)
**PND 90**									
*n* =	10	11	12	10	14	13	8	11	9
Distended glands	**7 (70%)**^a^	**7 (64%)**^a^	6 (50%)	**6 (60%)**^a^	**9 (64%)**^a^	**11 (85%)**^a^	**6 (75%)**^a^	**7 (64%)**^a^	4 (44%)
Cysts	0 (0%)	2 (18%)	1 (8%)	1 (10%)	2 (14%)	0 (0%)	1 (13%)	0 (0%)	1 (11%)
Gland nest density (nest/mm^2^±SE)	0.03±0.02	0.02±0.01	0.08±0.03	0.02±0.01	0.11±0.04	0.13±0.05	0.01±0.01	0.03±0.03	0.09±0.06

a *P*<0.05 by *χ*^2^ test for trend across PNDs in exposure group for increases in distended glands.
